# Gallstone sigmoid obstruction treated with electrohydraulic lithotripsy

**DOI:** 10.1055/a-2262-7914

**Published:** 2024-02-22

**Authors:** Jesus A. Guzman, Bhavi Trivedi, Mohammad Bashashati, Vital Rangashamanna, Brian Davis, Ihsan Al-Bayati, Sherif E. Elhanafi

**Affiliations:** 1 Division of Gastroenterology, Texas Tech University Health Science Center, El Paso, United States; 2 Department of Radiology, Texas Tech University Health Science Center, El Paso, United States; 3 Department of Surgery, Texas Tech University Health Science Center, El Paso, United States

Gallstone is a rare cause of large-bowel obstruction. It can result in complete mechanical obstruction in the setting of underlying distal benign or malignant narrowing 1 . Traditionally, surgery is considered the primary treatment modality but carries elevated morbidity and mortality, making nonoperative approaches such as endoscopic mechanical or electrohydraulic lithotripsy (EHL) an integral part of the management 2 .

A 79-year-old woman with a past medical history of uterine cancer and hysterectomy was found to have a large-bowel obstruction, secondary to a 3-cm gallstone impacted in the sigmoid colon from a fistulous communication between the gallbladder and colon at the hepatic flexure ( Fig. 1 ). Initial conservative management failed and the patient was deemed high risk for surgical intervention. Colonic decompression by stone extraction was attempted using conventional tools such as Roth net, snare, and rat-tooth forceps, but these efforts were unsuccessful. After a multidisciplinary discussion, the decision was made to proceed with repeat endoscopic decompression, with attempted fragmentation and removal of the gallstone using EHL.

**Fig. 1:**
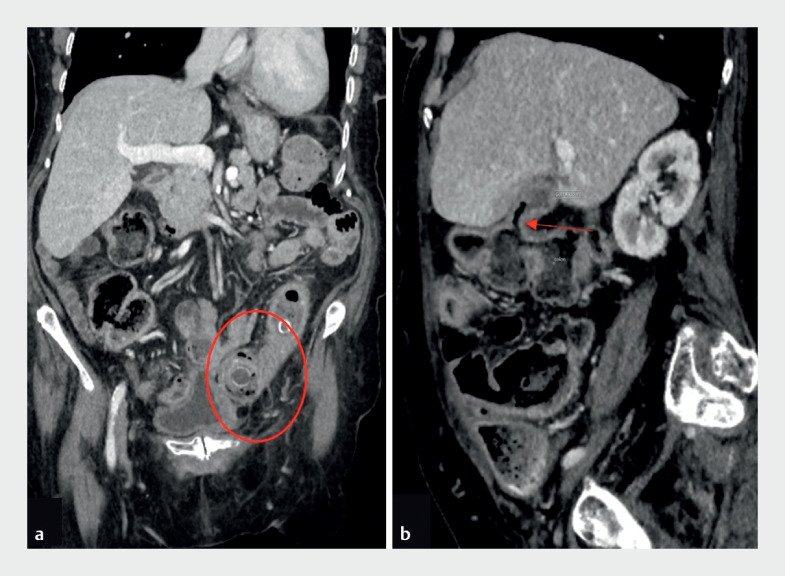
Computed tomography. **a** A 3-cm gallstone (red circle) was seen impacted in the sigmoid colon. **b** A fistulous communication (arrow) was evident between the gallbladder and colon at the hepatic flexure.

Colonoscopy revealed a large impacted pigmented stone in an area of peridiverticular colonic narrowing, causing significant ulceration and superficial necrosis of underlying mucosa. A 1.9-Fr EHL probe was passed through the biopsy channel of the gastroscope, and continuous normal saline irrigation provided a medium for EHL ( Video 1 ). EHL was performed using a Northgate Autolith Touch generator (Northgate Technologies, Inc., Elgin, Illinois, USA), using low power and frequency, which was increased in a stepwise fashion. After successful fragmentation, the scope was advanced beyond the area of obstruction, revealing a dilated colon and no evidence of perforation ( Fig. 2 ). A Jagwire (Boston Scientific, Marlborough, Massachusetts, USA) was introduced for the placement of a rectal tube. Overnight, the patient had multiple bowel movements and was then successfully discharged from the hospital.

Gallstone sigmoid obstruction treated with electrohydraulic lithotripsy.Video 1

**Fig. 2:**
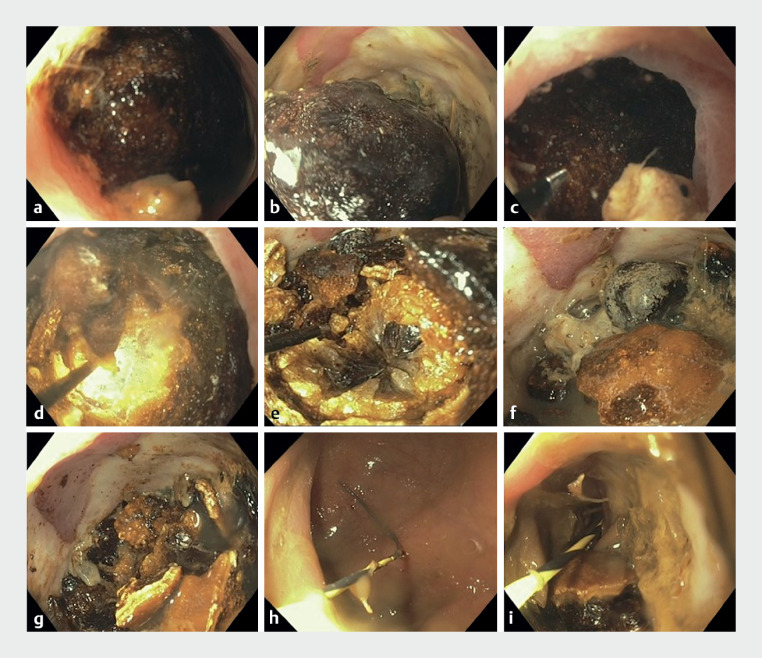
Endoscopic sequence of gallstone treatment with electrohydraulic lithotripsy (EHL). **a,b** The large, 3-cm gallstone was seen impacted in the sigmoid colon, causing significant ulceration ( **a** ) and superficial necrosis of underlying mucosa ( **b** ). **c–g** A 1.9-Fr EHL probe was passed through the biopsy channel of the gastroscope and EHL was performed to fragment the gallstone. Continuous normal saline irrigation provided a medium for EHL. **h,i** A Jagwire (Boston Scientific, Marlborough, Massachusetts, USA) was introduced for the placement of a rectal tube.

This case highlights the feasibility and safety of endoscopic management of large-bowel obstructions secondary to gallstones using lithotripsy modalities such as EHL, which could be considered a first-line therapeutic option.

Endoscopy_UCTN_Code_TTT_1AQ_2AF

## Notes

Endoscopy E-Videos https://eref.thieme.de/e-videos
*E-Videos* is an open access online section of the journal *Endoscopy* , reporting on interesting cases and new techniques in gastroenterological endoscopy. All papers include a high-quality video and are published with a Creative Commons CC-BY license. Endoscopy E-Videos qualify for HINARI discounts and waivers and eligibility is automatically checked during the submission process. We grant 100% waivers to articles whose corresponding authors are based in Group A countries and 50% waivers to those who are based in Group B countries as classified by Research4Life (see: https://www.research4life.org/access/eligibility/ ). This section has its own submission website at https://mc.manuscriptcentral.com/e-videos .
